# Supplementary material for: Segatella copri Outer-Membrane Vesicles Are Internalized by Human Macrophages and Promote a Pro-Inflammatory Profile
Source: Int J Mol Sci. 2025 Apr 11;26(8):3630. doi: 10.3390/ijms26083630 (PMC12027123; doi:10.3390/ijms26083630)
Supplement: Supplementary file 1 [file ijms-26-03630-s001.zip › ijms-3532266-supplementary.pdf]

Supplementary figure S1

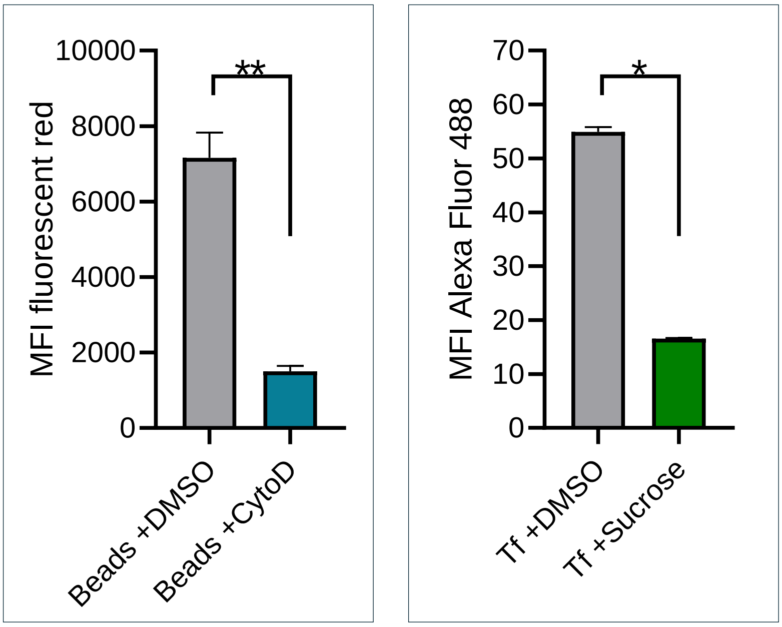

(a) (b)

Figure S1: Positive controls for inhibition of endocytotic pathways. THP-1-derived macrophages were preincubated for 1 hour with (a) 1 µg/mL cytochalasin D (CytoD), an inhibitor of phagocytosis/macropinocytosis, (b) 45 mM sucrose, an inhibitor of clathrin-mediated endocytosis, or vehicle (DMSO), before addition of (a) fluorescent red-labeled latex beads (Beads) for further 3 hours, or (b) Alexa Fluor 488-labelled transferrin (Tf) for 15 minutes. Mean ± SEM of (a) fluorescent red (n=3) and (b) Alexa Fluor 488 (n=2) mean fluorescent intensity (MFI) are displayed. Statistical significance was determined by paired t-test with *p < 0.05 and **p < 0.01.

Supplementary figure S2

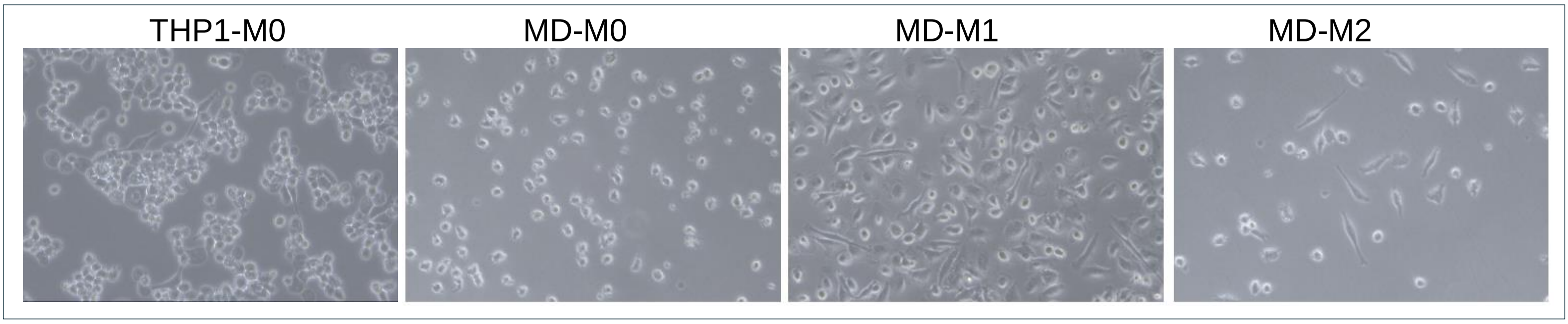

(a)

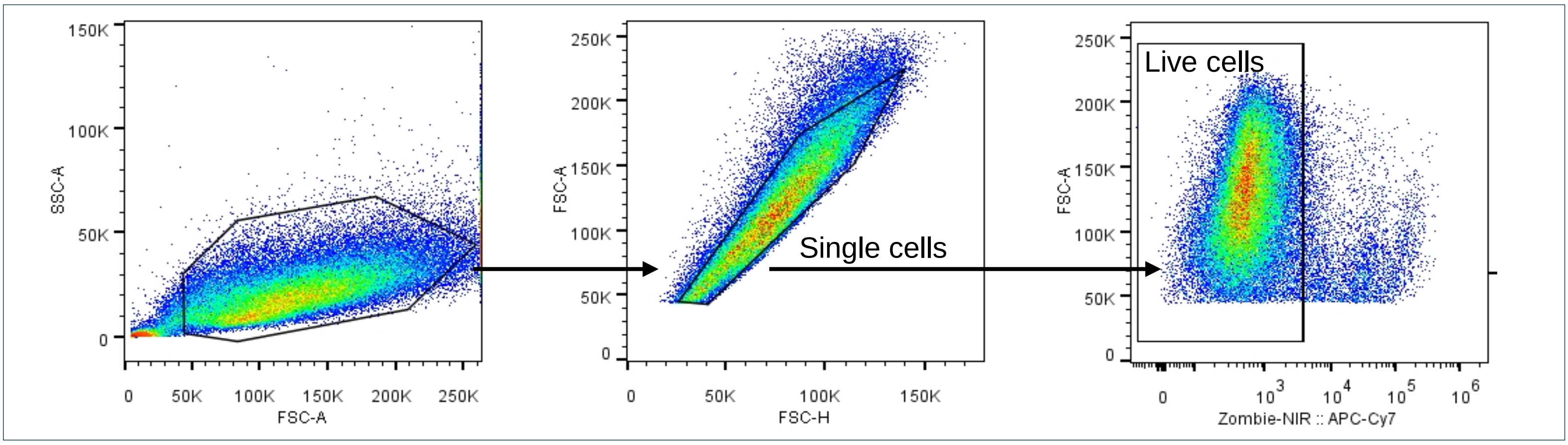

(b)

Figure S2: Macrophage morphology and gating strategy used for phenotypic analysis by flow cytometry. (a) Representative light microscopy images of macrophages obtained from monocytic THP-1 cell line (THP1-M0) and peripheral blood monocyte-derived (MD)-M0, M1 and M2 macrophages. (b) Gating strategy for the phenotypic analysis of human macrophages. The population of interest was identified by size and granularity using Forward and Side Scatter (FSC and SSC), then, the singlet cell population was selected, followed by gating on live cells (negative for the Zombie-NIR dye).

Supplementary figure S3

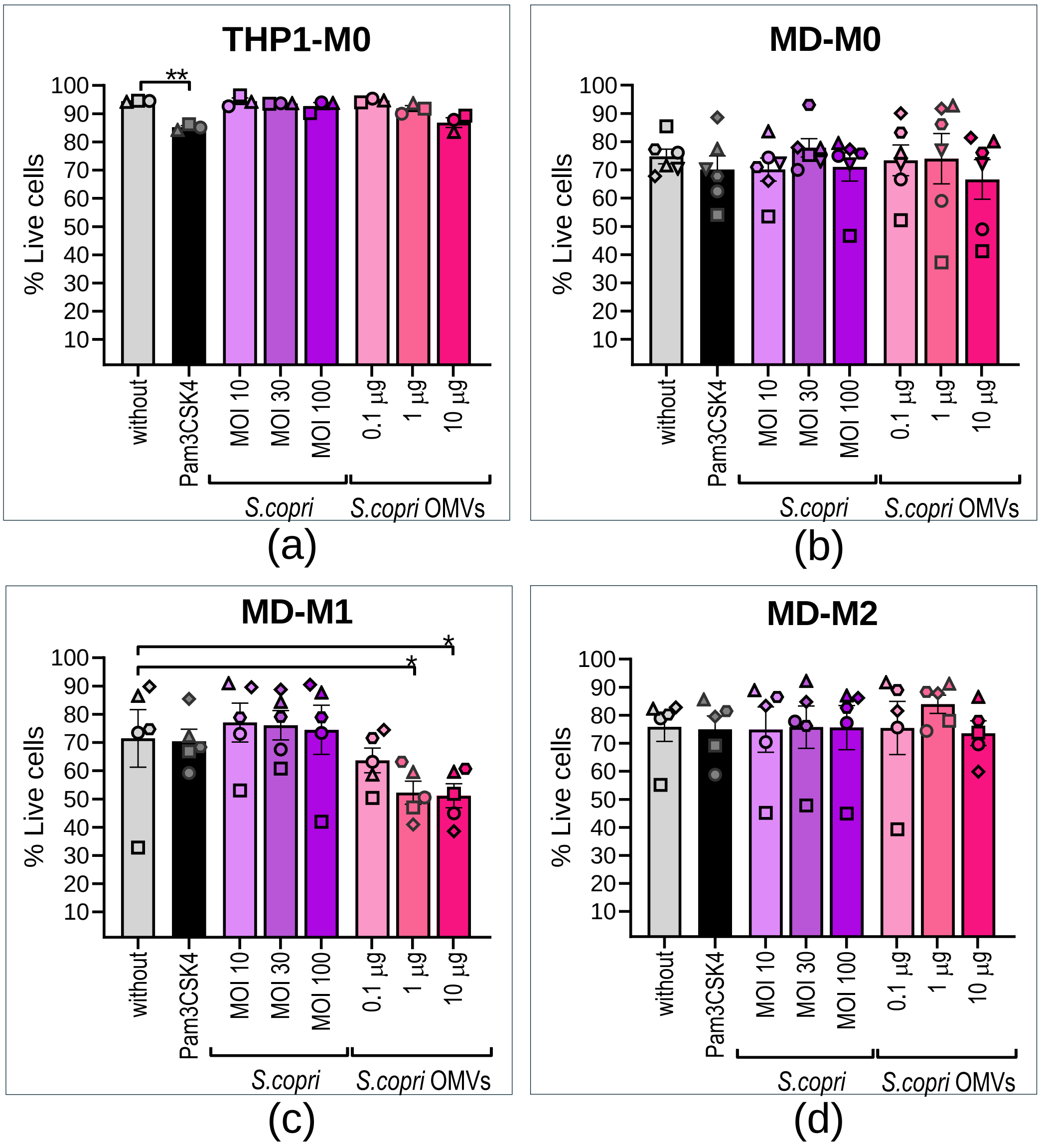

Figure S3: Viability of human monocyte-derived macrophages exposed to *S. copri* and its OMVs. Percentage of live cells was determined by exclusion of the viability dye Zombie-NIR within the THP-1-derived macrophages (a), as well as monocyte-derived M0 (b), M1 (c), and M2 (d) macrophages, which were stimulated for 24 hours with Pam3CSK4 (0.5 µg/mL), *S. copri* at a MOI of 10, 30, and 100, and OMVs at concentrations of 0.1 µg/mL, 1 µg/mL, and 10 µg/mL. Statistical significance was analyzed using one-way ANOVA and Dunnett´s post test with *$p \leq 0.05$ and **$p \leq 0.01$ (n=3-6).

Supplementary figure S4

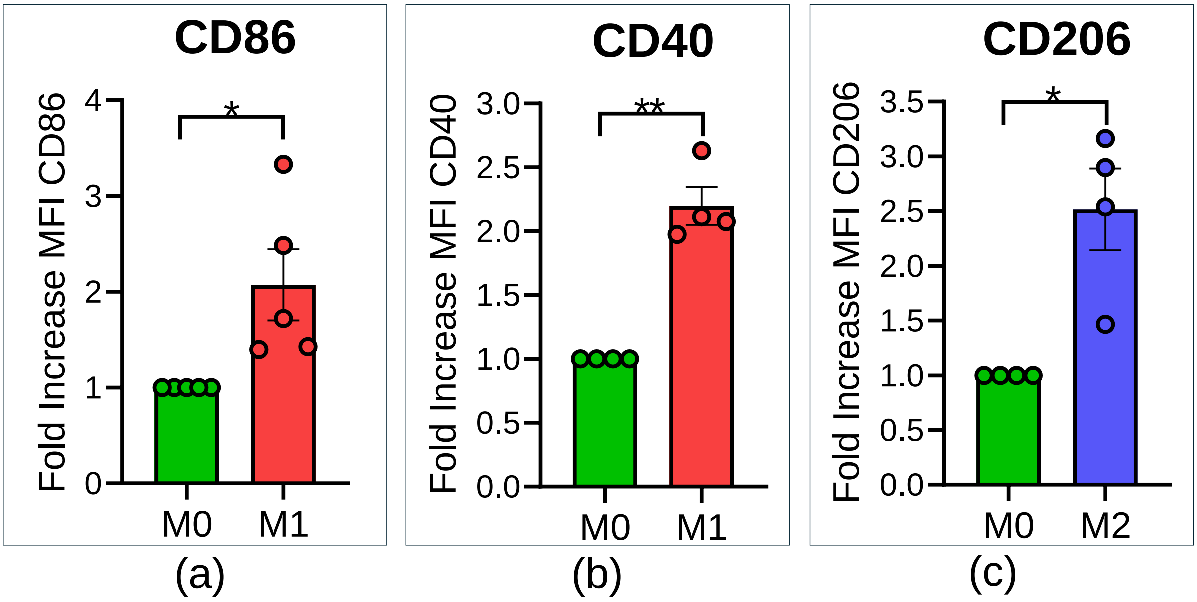

Figure S4: Confirmation of M1 and M2 polarization of monocyte-derived macrophages *in vitro*. Macrophages differentiated from peripheral blood monocytes and polarized into M1 in the presence of GM-CSF and IFN-γ (a,b) or M2 in the presence of M-CSF and IL-4 (c) show increase in markers associated with the respective profile: CD86 (a) and CD40 (b) for M1 and CD206 (c) for M2 with respect to naïve unpolarized M0 macrophages. Increase of mean fluorescent intensity (MFI) with respect to the unstimulated control were displayed as fold increase. Results were expressed as mean ± SEM. Statistical significance was determined by Mann-Whitney test with *$p < 0.05$ and **$p < 0.01$ (n=4).
